# A qualitative exploration of policy interventions to improve the health-related quality of life of people living with HIV AIDS and co-morbidities of hypertension and/or diabetes in Ghana

**DOI:** 10.1371/journal.pone.0311994

**Published:** 2024-10-11

**Authors:** Richmond Owusu, Serwaa Akoto Bawua, Emmanuel Bugyei Kwarteng, Leonard Baatiema, Justice Nonvignon

**Affiliations:** 1 Department of Health Policy, Planning & Management, School of Public Health, University of Ghana, Accra, Ghana; 2 Department of Biological, Environmental, and Occupational Health, School of Public Health, University of Ghana, Accra, Ghana; Federal Teaching Hospital, Ido-Ekiti, NIGERIA

## Abstract

**Introduction:**

The intersection of infectious diseases, such as HIV, with chronic conditions like hypertension and diabetes poses a significant challenge in global health. While advancements in antiretroviral therapy have transformed HIV into a manageable chronic condition, a growing number of individuals with HIV now grapple with coexisting non-communicable diseases, impacting their Health-Related Quality of Life (HRQoL). Despite strides in HIV care, there is a notable policy gap that undermines efforts to address HIV-associated co-morbidities, particularly hypertension and diabetes, especially efforts to improve access, early detection, and ultimately HRQoL for individuals with HIV and co-morbidities. This study seeks to explore policy interventions aimed at improving the quality of life of HIV patients with hypertension or diabetes.

**Methods:**

The study utilized a qualitative descriptive design to explore the experiences and perspectives of healthcare professionals and support staff regarding policy interventions for managing HIV patients with hypertension and/or diabetes co-morbidities in three regions of Ghana. The research was conducted in the Upper West, Ashanti, and Greater Accra regions among 11 participants, chosen purposively from professions involved in HIV patient care to understand their views on the implementation of policy interventions to HRQoL for individuals with HIV and co-morbidities. In-depth interviews were conducted face-to-face and tape-recorded. Thematic analysis approach was used to analyze the data.

**Results:**

The study involved 11 participants from three regions with varied years of experience. Implemented policies that potentially improve the HRQoL for individuals with HIV and co-morbidities involve support groups, home visits, provision of free drugs, and counselling. Barriers to policy implementation included non-adherence to medication, stigma, cost of non-communicable diseases (NCDs) medications, accessibility issues to NCDs services, lack of interest or understanding among implementers, and high staff turnover. Facilitators encompassed in-service training, guidelines in common platforms, knowledge sharing, external resources, regular check-ups, and motivational packages for patients.

**Conclusion:**

Individuals with HIV and comorbidities face complex challenges impacting their HRQoL, including emotional and financial dimensions. The study identifies critical policies and barriers, underscoring the need for tailored, patient-centered approaches. Facilitators like in-service training and regular check-ups offer actionable insights for effective policy implementation, emphasizing improved health outcomes for those with comorbid conditions. The study recommends integrated care approach and adherence support programs that address the unique challenges faced by people living with HIV.

## Introduction

The global burden of diseases, particularly Human Immunodeficiency Virus (HIV) and non-communicable diseases (NCDs) such as hypertension and diabetes, represents a significant challenge for public health systems worldwide. These conditions not only pose a threat to individual health but also strain healthcare resources and impact the overall socioeconomic development of nations [1]. HIV, a virus that attacks the immune system, has been a major global health concern for decades. According to the World Health Organization (WHO), as of the latest available data in 2023, approximately 39 million people were living with HIV worldwide [2]. The WHO facts sheet in 2023 estimates that NCDs account for a significant proportion of the global burden of disease, contributing to approximately 74% of all global deaths [3]. In low- and middle-income countries (LMICs), NCDs accounts for a large proportion of all mortalities [4, 5]. The burden of NCDs is high and expected to increase in LMICs, with deaths due to NCDs projected to rise substantially by 2030 [6]. In comparison, HIV/AIDS receives a significant amount of global health funding, while NCDs receive a disproportionately low amount [7]. The prevalence and incidence of NCDs among adults living with HIV in sub-Saharan Africa (SSA) is of particular concern, as SSA accounts for a large proportion of people living with HIV globally [8].

Additionally, individuals with HIV and comorbidities such as hypertension or diabetes face complex health challenges. According to Baker et al. 2018 and Nduka et al. 2018, the presence of hypertension may elevate the risk of cardiovascular complications, as hypertension is a known risk factor for heart disease and stroke [9, 10]. Similarly, individuals with comorbid HIV and diabetes may experience an increased likelihood of cardiovascular complications, given the elevated cardiovascular risk associated with diabetes [11, 12].

Beyond cardiovascular complications, the coexistence of HIV and these NCDs may impact the overall progression of HIV. Studies have indicated that comorbidities, particularly hypertension, may contribute to immune dysregulation and potentially influence the course of HIV infection [13, 14]. Managing both HIV and NCDs proves challenging, with potential interactions between antiretroviral drugs and medications used for conditions like hypertension or diabetes, necessitating careful monitoring and treatment adjustments [15]. Furthermore, the presence of multiple chronic conditions can significantly impact the overall quality of life for individuals with HIV, affecting daily living and potentially leading to increased mortality risk compared to those without comorbid conditions [16–18]. Disparities in access to healthcare further compound these challenges, emphasizing the crucial need for integrated and comprehensive healthcare strategies for individuals dealing with dual burden of HIV and NCDs [19–21].

Despite significant strides in HIV care, there exists a noticeable gap in policies specifically tailored to address the co-morbidities associated with HIV, particularly hypertension and diabetes [22, 23]. Policy interventions play a pivotal role in shaping the healthcare landscape, offering a framework to enhance access, promote early detection, and ultimately improve HRQoL for individuals with HIV and co-morbidities [24, 25]. Different policy interventions have been implemented to improve the HRQoL for HIV patients living with co-morbidities of hypertension and/or diabetes. These include multilevel interventions, long-term care, rehabilitation, mental health support, behavioral therapy, and social support [26]. Furthermore, these interventions aim to integrate care for HIV and NCDs and empower patients to manage their conditions effectively. Patient perspectives on integrated care have been mostly positive, with reduced stigma, travel and treatment costs, and a more holistic approach to healthcare [27]. However, challenges such as long waiting times and a lack of continuity of care have been identified [28]. Interventions targeting multiple levels, including patient-level, organizational, and political barriers, have been found to be more effective than single-level interventions [29]. Additionally, lifestyle interventions such as physical activity and aerobic exercises have shown potential in reducing cardiovascular conditions and improving quality of life among HIV patients [30]. In Ghana, these interventions include the development of overarching policies and strategies, although challenges such as funding and disproportionate focus on communicable diseases have been identified. Social support has been found to be a key factor in improving the quality of life for HIV patients [31], while stigma reduction interventions have also been highlighted as important [24]. However, food and nutrition assistance for HIV-infected individuals in Ghana remains inadequate [32].

There are other challenges in successfully implementing HIV treatment policy in Ghana, including individual and environmental factors that hinder policy implementation [33]. Also, to monitor the accuracy of HIV rapid testing results, a national proficiency testing program using dried tube specimens was rolled out in Ghana, leading to improved testing performance rates [34]. Policymakers and healthcare providers in Ghana express support for the introduction of oral pre-exposure prophylaxis (PrEP) for key populations, but have concerns about behavioral disinhibition, non-adherence, and cost [35]. The HIV/AIDS education program in the Lower Manya Krobo Municipality of Ghana faces challenges due to conflicting policies promoting abstinence and comprehensive sex education, highlighting the need for realignment of policies to ensure transparent implementation of HIV/AIDS sex education programs [36]. Interventions such as the adaptation of the Many Men Many Voices program using the ADAPT-ITT model have been implemented to address the needs of men who have sex with men (MSM) in Ghana, emphasizing cultural relevance and collaboration with community partners [37].

These studies have not provided any specific policy interventions designed to address the management of NCDs in HIV patients, despite the growing prevalence of such conditions among this population, this study seeks to explore and evaluate the existing policy interventions aimed at improving the quality of life of HIV patients with hypertension or diabetes.

## Methods

### Study design and setting

The study employed qualitative descriptive design. Phenomenology approach was used to understand the experiences, perceptions, and opinions of healthcare professionals and support staff regarding existing policy interventions for managing HIV patients with co-morbidities of hypertension and/or diabetes. The study was conducted in three regions of Ghana, encompassing the Savannah, middle belt, and coastal belt ecological zones, more precisely in the Upper West, Ashanti, and Greater Accra regions respectively to ensure representation of perspectives from the coastal, middle and northern belt of Ghana. These regions were purposively selected due to the high prevalence of HIV cases in their respective ecological zones, as reported in the latest Ghana AIDS Commission fact sheets from 2019 [38]. Wa Municipal Hospital, Kumasi South Hospital and Madina Polyclinic were purposively selected as the sites for conducting the qualitative interviews. These health facilities are well established healthcare facilities that provide antiretroviral therapy and other essential services like chronic care. Additionally, these facilities are situated within the capital of the selected regions and therefore have large population of HIV patients and also serve as a referral point for ART services for other facilities within their respective regions.

### Study population, sample size and sampling

Healthcare professional and support staff directly involved in the care and management of HIV patients were included in the study. Inclusion in the study involved health professionals who possess a minimum of two years of experience within the ART unit. Health personnel of a non-permanent nature and those who declined to offer informed consent for participating in interviews were excluded from the study. With the aid of a purposive sampling, 11 participants were selected from the three regions. Selected participants were taken through the informed consent process after they had studied the participant information sheet developed for the study.

### Data collection

Using a pre-tested interview guide, in-depth interviews were conducted among frontline healthcare professionals and support staff for HIV-NCD treatment services from 24^th^ August to 26^th^ September 2023. The information collected from the respondents included background data, experience and knowledge of policy interventions aimed at improving the quality of life of patients living with HIV and NCDs. The interviews were done face-to-face, and it was tape recorded. The interviews were carried out by one of the authors, who holds a master’s degree in health economics and has been a research assistant for nearly three years, with relevant training and experience in conducting qualitative interviews.

### Data analysis

The qualitative in-depth interviews were analysed through a thematic approach. Thematic analysis is a flexible and accessible method for identifying, analyzing, and reporting patterns within data, providing a rich and detailed account of the data. In thematic analysis, it is important to conduct a thorough and comprehensive coding process, ensuring internal coherence and consistency of themes, and providing a convincing and well-organized story about the data and topic [39]. In this study, audio data were transcribed and ensured data was organized, then initial coding was identified to capture the key ideas and concepts within the data. Themes were developed and interpreted through organization of the initial codes into broader themes or patterns that capture the overall meaning of the data. Themes were then reviewed and revised to ensure data was accurately captured and there was coherent. Themes were interpreted, and insights were developed. Finally, findings were presented in a clear and concise manner.

### Trustworthiness

Member checking was employed to validate findings with participants, ensuring the accuracy and authenticity of their perspectives. Peer debriefing involved seeking feedback from colleagues with qualitative research expertise, enriching the study’s credibility. The use of quotations from participants adds depth and authenticity to the research. Additionally, the inclusion of external perspectives through peer review and the application of triangulation techniques further confirms the study’s findings, collectively enhancing its trustworthiness.

### Ethical considerations

Ethical clearance was obtained from the Ghana Health Service Ethics Review Committee with reference number: GHS-ERC027/05/23. All participants were requested to grant their consent in order to participate in the interview, and the entirety of the gathered data was rendered anonymous.

## Results

Table 1 shows the demographic profile of the participants. A total of 11 healthcare professionals and support staff were recruited from the three regions, out of this, 4 were from Greater Accra region, 4 from Ashanti region and 3 were from the Upper West region. Regarding experience, the minimum years of experience was 3 years and maximum was 18 years.

**Table 1:** Demographic profile of participants (in-depth interviews).

Participant’s ID Number	Sex	Region	Professional Background	Years of Experience
G-R1	Female	Greater Accra	Principal Nursing Officer	16 years
G-R2	Male	Greater Accra	Nurse	5 years
G-R3	Female	Greater Accra	Enrolled Nurse	5 years
G-R4	Female	Greater Accra	Staff Nurse	11 years
A-R1	Female	Ashanti	Principal Nursing Officer	10years
A-R2	Male	Ashanti	Data Manager	5 years
A-R3	Female	Ashanti	Nurse	5 years
A-R4	Male	Ashanti	Nurse	3 years
W-R1	Male	Upper West	Biostatistician	18 years
W-R2	Female	Upper West	Human Resource	3 years
W-R3	Male	Upper West	Research Officer	8 years

### Summary of themes

The study identified three overarching themes related to policies and interventions aimed at improving the quality of life for People Living with HIV/AIDS (PLWHA) with comorbidities of hypertension and/or diabetes. Firstly, the theme on "Policy and Interventions" explored strategies such as support groups, home visits, provision of free drugs, and counseling, implemented at the national level to enhance the health-related quality of life for PLWHA with comorbidities. Secondly, the theme addressing "Barriers to Implementation" highlighted challenges like non-adherence to medications, stigma, cost concerns, accessibility barriers, and staff-related issues that impede the effective execution of these policies. Lastly, the theme focusing on "Facilitators to Implementation" unveiled positive factors such as in-service training, common platform guidelines, knowledge sharing, external resources for updates, regular check-ups, and motivational packages that act as facilitators in overcoming barriers and supporting the successful implementation of policies for PLWHA with hypertension and/or diabetes comorbidities.

#### Theme 1: Policy and interventions

An analysis of the data from the participants’ interviews revealed four sub-themes of national level policies and interventions to improve the health-related quality of life of HIV patients living with co-morbidities of hypertension and/or diabetes and these comprised a) a support group system b) home visits c) provision of free drugs and d) counseling.

*Sub-theme 1*: *Support group system*. This policy involves the establishment and facilitation of support groups for individuals living with HIV and co-morbidities. Support groups provide a platform through regular meetings for patients to share experiences, challenges, and coping strategies. It fosters a sense of community and mutual understanding among patients, contributing to their emotional well-being and mental health.

*“We have the viral suppression. So we have adherence. We have support group system. So support group that we have meetings”. (G-R1)*

“And with the diabetes too, usually we have a support group meeting that will usually hold every month, so with this most of these things we talk about them, especially with their diets. We educate them on what they need to eat. We have nutritionists who also come around to also guide them on what to eat and not to eat, and the time to also eat so that it will not end up causing an increase or a rise in maybe your BP levels and your sugar" (G-R2)

*" Oh*, *for here*, *we do one monthly support group meeting for them here too and every month comes with its topic and from every department*, *we have a doctor coming some months*, *the lab*, *pharmacy*, *social worker and we talk to them*. *" (G-R3)*

*Sub-theme 2*: *Home visits*. The policy of conducting home visits extends healthcare services directly to the homes of patients. Home visits enable healthcare professionals to assess the living conditions, medication adherence, and overall health status of patients in their familiar environment, leading to more personalized and holistic care.

*“And even some of, all of them, we have times within the month that we even visit them at home to speak with them, know their challenges, and also advise them also on what to always eat to help.” (G-R2)*

*“We have home visiting so we do them and then we have counseling*. *And then there are lots*. *So these are the ones that come to mind*.*” (G-R1)*

*Sub-theme 3*: *Provision of free drugs*. Ensuring the availability of essential medications without financial burden is a critical policy to enhance the health-related quality of life for HIV patients with co-morbidities. This sub-theme emphasizes the commitment to providing antiretroviral at no cost to the patients. Financial constraints often hinder access to necessary medications, and this policy aims to alleviate such barriers, promoting consistent and uninterrupted treatment.

*" So, for now, what I know is for the ARVs, they take it for free. And the other ones we write for them, that’s the sceptrin and the Iron capsules they buy it themselves." (G-R3)*

The policy of providing free drugs aims not only to alleviate the financial burden of patients but also to enhance adherence.

*“You see our treatment is free, so they have free medication. And then with the adherence, the medication type is three medications combined to one and you take it only once a day” (G-R1)*

*Sub-theme 4*: *Counseling services*. The policy of counseling focuses on providing psychological and emotional support to individuals dealing with HIV and co-morbidities. Counseling services intend to address stigma, fear, and anxiety associated with these health conditions. It also plays a crucial role in educating patients about the importance of treatment adherence, lifestyle modifications, and coping strategies.

*“…and then we have counseling. And then there are lots. So these are the ones that come to mind.” (G-R1)*

Individuals facing stigma and discrimination are connected to a paralegal who engages with a psychosocial counselor to provide assistance and support.

*“If they are going through stigma and discrimination, we also have to link them to the paralegal. The paralegal will also talk to a psychosocial counselor. And they will also come in and assist.” (A-R1)*

#### Theme 2: Barriers to implementation of the policies to improve quality of life of PLWHA and hypertension/diabetes comorbidities

This theme explores the challenges and obstacles encountered in the practical application of national-level policies designed to enhance the well-being of individuals dealing with both HIV/AIDS and co-existing hypertension or diabetes. The theme reveals various impediments that hinder the effective implementation of these policies, shedding light on critical barriers that need attention for successful healthcare delivery. These challenges were peculiar to each region.

*Sub-theme 1*: *Non-adherence to hypertension and diabetes medication/pill burden*. This sub-theme underscores the challenge of individuals not consistently following prescribed medication regimens for hypertension and diabetes, which, in turn, hinders the successful implementation of policies aimed at enhancing the QoL for HIV patients with co-morbidities. This non-adherence results from various factors, including the burden of having to take multiple medications, often referred to as pill burden. Individuals with HIV who also have hypertension and/or diabetes may find it challenging to adhere to complex medication schedules, leading to suboptimal health outcomes. This challenge was reported in the Greater Accra region and the Ashanti region.

*"So usually here, what we do is when you come, we check your BP first of all because at times some of them, they feel that since I’m on my ARVs, I don’t have to even take any other drug, so you see people who are hypertensive, so they’ll think that this ARV will work everything out for them" (G-R2)*

Patients facing HIV along with hypertension and diabetes may experience a substantial pill burden, as they are required to take antiretrovirals, hypertensive drugs, and diabetic medications, contributing to the complexity of their medication regimen.

*"So, such a patient will have a high drug pill burden because the person will have to swallow the ARVs, will have to swallow the hypertensive drugs, will have to swallow the diabetic drugs…"(A-R2)*

*Sub-theme 2*: *Stigma*. Stigma, whether from society or self-imposed, creates a hostile environment that discourages individuals from seeking and adhering to necessary healthcare interventions. This challenge was reported across the three regions. Respondents from the three regions emphasized stigma as one of the biggest challenges faced in policy implementation.

*"I think most of the issues could be stigma which could be self-stigma…" (W-R1)*

*"Yeah*, *the experience*, *let me say*, *majority of them are self-stigma*. *Self-stigma is one of their major challenge because immediately they come and see a new face*. *You know*, *they have been here for a long*. *So*, *when they come and see a new face*, *the stigma that is always on them is higher*. *So*, *I think that is the major challenge facing those people*. *The stigma*… *your relatives will come*… *and see you*, *I have an experience*, *one of my aunties came and when she saw me*, *she couldn’t come inside to collect the medication*.*" (W-R2)*

Respondents also reported that individuals not only face self-stigmatization but also project stigma onto healthcare providers, presenting a significant challenge in the context of managing HIV and comorbidities.

*“Stigma. Stigma has always been the challenge. They stigmatize themselves, then stigmatize us."(A-R3)*

*Sub-theme 3*: *Cost of NCDs medications*. Across the three regions, financial burden associated with obtaining necessary medications for these NCDs was reported to pose a significant challenge for HIV individuals. The high cost of hypertensive and diabetic drugs may lead to financial strain, making it difficult for patients to afford these essential drugs consistently.

*“And mostly when it comes to HIV, you see that our care is sort of free. But with theirs, they have to pay. So, burden, they don’t understand. They are used to the free system, coming for medication, now they have diabetes and they have to buy their own drugs and stuff. So, it’s quite difficult for them financially.” (G-R1)*

*Sub-theme 4*: *Accessibility barriers to NCDs services*. Accessibility barriers encompass obstacles that impede individuals’ ability to readily obtain healthcare services and resources for other co-morbid conditions. This obstacle was reported in the Greater Accra and Upper West regions, as patients facing the dual burden of HIV and co-morbid conditions encounter difficulties in accessing the necessary healthcare services due to the unavailability of these services at the ART units.

*“so what we do is to do referral and we refer the HIV patients with diabetes or hypertension to those departments for those services to be continued there so in actual fact there is no direct link to us if the person gets there… In actual fact, we were supposed to be doing some of the tests here, but logistics constraints and the specialists involved, it’s always a challenge.” (W-R1)*

Respondents stated the logistical challenges faced in healthcare facilities, specifically addressing space limitations, which result in a fragmented patient experience where individuals may need to move between different locations for various aspects of their care, including medication dispensing, consultations, and administrative tasks.

*“…for instance, for here, you know, we lack space. So, at times you need to move the client from one place to another. Especially when they are coming for their medications, for instance. So, you need to attend to the person over here, then the patient will go elsewhere, pick a folder, see the doctor and take his/her medications.” (G-R2)*

*Sub-theme 5*: *Lack of interest or understanding among implementers*. This barrier pertains to a situation where individuals responsible for executing and applying the policies lack sufficient interest or understanding in the goals and mechanisms of these interventions. It suggests that a lack of commitment, awareness, or comprehension among healthcare providers and other implementers may impede the successful execution of policies. This lack of interest or understanding can result in suboptimal adherence to prescribed protocols, inadequate communication with patients, and an overall diminished impact of the intended interventions. This barrier was reported in the Upper West region.

*“……the implementers could also be a challenge. So, the person who is supposed to implement it, if he’s not interested in that policy, forget about it. Yeah, forget about it. It will hang like a skeleton and no flesh will come on it.” ….that’s what I said on the staff side could be a challenge”(W-R1)*

*Sub-theme 6*: *High staff turnover and continuity*. This barrier was reported in the Upper West region and involves the frequent turnover of staff members responsible for implementing these policies, leading to challenges in maintaining continuity and consistency in the delivery of healthcare services. High staff turnover can disrupt the implementation process, as new staff may require time to familiarize themselves with the policies, protocols, and patient populations.

*“And those interventions have been put in place, but as we all know, staff attritions and the rest, people are leaving*… *For instance, ANC was supposed to be giving medication at ANC but after training the staff, they were moved, so now the new people cannot also give medication.”*(*W-R1)*

#### Theme 3: Facilitators to the implementation of the policies to improve quality of life of PLWHA and hypertension/diabetes comorbidities

This theme encapsulates the factors that support the successful execution of policies aimed at enhancing the health-related quality of life for individuals living with HIV and co-morbidities of hypertension and/or diabetes. Facilitators within this theme contribute to overcoming challenges and promoting the effective implementation of policies. These facilitators include in-service training and workshops, guidelines in common platforms, knowledge sharing, external resource for updates, regular check-ups, and motivational packages for patients.

*Sub-theme 1*: *In-service training and workshops*. In-service training and workshops play a crucial role in equipping healthcare professionals and support staff with updated knowledge, skills, and insights related to the management of co-morbid conditions. These training sessions provide a platform for disseminating information about new policies, interventions, and best practices, ensuring that healthcare providers stay informed about the evolving landscape of HIV and comorbidity care. By enhancing the expertise of the healthcare workforce, in-service training and workshops contribute to improved patient care, effective implementation of policies, and ultimately, better health outcomes for individuals dealing with the complexities of HIV and co-existing conditions. This was reported in all the three regions as a facilitator to effective implementation of policies and interventions.

*"Most of the time is through in-service training. Within this month, I’ve attended four trainings. Two on gender-based violence. We met DOVVSU, we met CHRAJ, we met the psychiatrist, we met lawyers, and they discussed how we should manage cases like rape, assault and other things." (A-R1)*

*"Yes*, *we call it the continuous training*.*" "*…*in-service trainings where two or three people go for the training come back and are expected to train the others on the ground*.*" (W-R1)*

Respondents highlighted the role of workshops as a valuable mechanism for disseminating information and knowledge about healthcare policies.

*"There are a lot of workshops that come in….So I think it’s through some of these things some of us also know the policies."(W-R2)*

*Sub-theme 2*: *Guidelines in common platforms*. Having guidelines accessible through common platforms provides a standardized and easily accessible reference for healthcare professionals and support staff involved in the care and management of individuals with co-morbid conditions. Common platforms such as WhatsApp platforms, healthcare information systems, or shared documents that ensure uniformity and consistency in the application of policies. This accessibility to guidelines streamlines decision-making processes, promotes adherence to established protocols, and fosters a cohesive approach to managing the complexities of HIV and comorbid conditions.

*"We have a platform where whenever there are new policies, things that we need to implement, it goes through the districts…" (G-R2)*

*“So*, *for me*, *mostly*, *when it comes to updating myself*, *we have these platforms that we are on*. *Okay*, *where the program managers and then the other officers at the national level*, *they put on stuffs” (W-R3)*

*Sub-theme 3*: *Knowledge sharing and utilizing*. This sub-theme involves creating an environment where healthcare professionals and support staff actively share information, experiences, and insights related to the care and management of individuals with these co-morbid conditions. Through knowledge sharing, best practices and successful strategies can be disseminated among the healthcare community, promoting a collaborative and informed approach to patient care. Additionally, utilizing shared knowledge ensures that evidence-based information is applied consistently, contributing to better decision-making and more effective implementation of policies.

*" I went for the training and when I came back what I did was, we went with the district, so what we did was after we returned from the training, we did a step down like debriefing we did the debriefing to the facility and the district people as well and then the ART team, we actually debriefed them and we showed them the way forward and all the reporting tools… "(A-R2)*

*“When there’s a training*, *we go for the training and then we come to teach the rest to continue the process*.*” (W-R1)*

*Sub-theme 4*: *External resources for updates*. External resources play a crucial role in providing additional information, research findings, and updates on best practices and interventions. By accessing external resources, healthcare professionals and support staff can stay abreast of the latest developments in the field, including advancements in treatment options, emerging research, and changes in healthcare policies.

*“…so because we have the internet as well, and there are some series on HIV, like South Africa, they have a series which they show, and they talk about more of these because South Africa is one of the countries leading in HIV so sometimes I follow those things just to be abreast with because they also mention some of these updates and stuff and it’s worth it.” (A-R2)*

*“But mostly I also go online to do my research to find out is there anything new coming from World Health Organization*.*” (W-R3)*

*Sub-theme 5*: *Regular check-ups on clients*. Regular check-ups play a crucial role in the monitoring and management of the health status of individuals with these conditions. These check-ups enable healthcare professionals to assess the effectiveness of interventions, the adherence level, track the progress of treatment, and identify any emerging health issues promptly.

*“So, when you are not able to come that day, we call you to check up on you and know why you couldn’t come on your date just to make sure you are not missing your drugs. When you travel, we encourage you to go to a nearest hospital to take up your drugs so that you don’t break the chain.” (G-R4)*

*Sub-theme 6*: *Motivational packages to patients*. Motivational packages play a pivotal role in encouraging and empowering individuals to adhere to prescribed treatments, lifestyle modifications, and overall healthcare regimens. These packages include various financial and non-financial incentives, rewards, or educational materials designed to motivate patients to actively engage in their health management.

*“…when they come for the meetings, we give them things. We give them refreshments and all that so some of them I’m sure because of that, that’s why the following month they come….. And yearly too we do a party for them at the end of the year and that one too we give awards for those who come for daily meetings, like throughout the year, the person who has been consistent, we give them awards and all that, so it motivates most of them.” (G-R3)*

Also, respondents highlighted the effort made healthcare providers to organize programs aimed at reducing stigma, where clients are taken for trainings and providing clients with opportunities for relaxation which not only contribute to their well-being but also serve as a means of support and encouragement, potentially motivating them to actively engage in their health management.

*“we have programs we organize for them so that they can get away that kind of stigma issues, so there are people we take them out for trainings, we take our clients out. They go to the hotels, spend some days, about three days, eat morning and evening after that they give them money…” (A-R4)*

## Discussion

The study’s findings illuminate the complex landscape surrounding policies for improving the health-related quality of life of People Living with HIV/AIDS (PLWHA) coping with comorbidities of hypertension and/or diabetes. National-level policies and interventions, encompassing support groups, home visits, provision of free drugs, and counseling, underscore a holistic approach. However, pervasive barriers such as non-adherence, stigma, financial constraints, and accessibility issues demand targeted interventions. The study emphasizes the pivotal role of healthcare providers and identifies facilitators, including in-service training, common guidelines, knowledge sharing, external resources, regular check-ups, and motivational packages, as crucial for effective policy implementation.

This theme delves into the national-level policies and interventions designed to enhance the quality of life for PLWHA dealing with hypertension and/or diabetes. The sub-themes, including a support group system, home visits, provision of free drugs, and counseling, highlight the multifaceted approach required to address the complexities of comorbid conditions. These policies have also been highlighted in the Guidelines for Antiretroviral Therapy in Ghana (2017) and National HIV and AIDS Policy (2019) [40, 41]. The support group system and home visits emerge as a crucial policy to address the emotional and psychological needs of PLWHA and allow healthcare professionals to assess living conditions, medication adherence, and overall health status in the familiar environment of patients, this was similar to the findings in Zimbabwe [42] and South Africa [43]. The provision of free drugs addresses financial barriers faced by PLWHA. As reported in the Guidelines for Antiretroviral Therapy in Ghana (2017) and National HIV and AIDS Policy (2019), ART in Ghana is free to all clients with valid National Health Insurance cover (NHIS), however absence of an NHIS card shall not be a barrier to treatment [40, 41]. This policy acknowledges the economic challenges individuals may encounter and emphasizes the commitment to providing essential medications without imposing a financial burden as reported by Yenet et al. (2023) [44]. By alleviating cost concerns, this aims to promote consistent and uninterrupted access to necessary medications, thereby supporting overall treatment adherence.

Additionally, counseling services emerge as a critical policy to address the stigma associated with HIV and comorbid conditions. According to the Guidelines for Antiretroviral Therapy in Ghana (2017), The goal of counselling is to help the client make an informed decision to start and to adhere to a life-long treatment [40]. This focuses on providing psychological and emotional support, emphasizing the importance of holistic care as reported by Remien et al. (2019) [45]. Counseling plays a pivotal role in educating patients about treatment adherence, lifestyle modifications, and coping strategies, contributing to a comprehensive approach in improving the quality of life for PLWHA dealing with comorbidities. These policies and interventions are seen as positively impacting the quality of life of patients, promoting adherence, improving psychological well-being, and aiding in viral suppression [46, 47].

Each region faces distinct challenges impacting the effective implementation of interventions. Non-adherence to medication regimens is identified as a barrier, particularly in regions where patients face challenges in following prescribed schedules, this finding was similar to a scoping review qualitative research by Kvarnström et al. (2021) [48]. Factors such as the burden of multiple medications and language barriers contribute to this challenge, highlighting the need for tailored interventions that consider individual patient needs, preferences, and socio-cultural contexts. Also, stigma, both self-imposed and societal, emerges as a pervasive barrier affecting PLWHA. The reported challenges across regions emphasize the urgent need to address stigma through comprehensive interventions, including educational programs, community engagement, and destigmatization campaigns.

Furthermore, financial constraints associated with obtaining medications for comorbid conditions pose a significant challenge, this is similar to the findings in Malawi [49] and United States [50]. The discussion underscores the financial burden faced by individuals, particularly when transitioning from a system where HIV treatment is free to one where they have to bear the cost of medications for other conditions. Addressing this barrier requires targeted strategies to ensure affordability and financial support.

Accessibility barriers highlight challenges in accessing necessary healthcare services, especially in regions where there is a lack of direct links between different departments. The reported logistical constraints underscore the importance of streamlining healthcare services, improving infrastructure, and enhancing coordination to ensure seamless access for individuals with comorbidities. In addition, lack of interest or understanding among implementers emphasizes the crucial role of healthcare providers and implementers in the successful execution of policies. Lack of interest or understanding among implementers can hinder effective policy implementation. Addressing this challenge necessitates targeted efforts, including training programs, awareness campaigns, and fostering a culture of commitment and understanding among healthcare professionals. Lastly, high staff turnover poses a challenge to the continuity and consistency of healthcare services [51]. The reported disruptions in the implementation process highlight the need for strategies to mitigate the impact of staff turnover, such as comprehensive onboarding programs, knowledge transfer mechanisms, and policies that prioritize staff retention.

These challenges highlight the intricate interplay between individual attitudes, institutional dynamics, and external factors in hindering effective policy implementation [52].

Distinct factors contribute to overcoming barriers and supporting successful policy implementation. In-service training and workshops emerge as crucial facilitators to enhance the knowledge and skills of healthcare professionals [53]. The reported attendance in multiple training sessions highlights the commitment to continuous education, ensuring that healthcare providers stay updated on evolving practices, policies, and interventions. This emphasizes the significance of investing in the professional development of healthcare professionals to improve the quality of care. Also, having guidelines accessible through common platforms provides a standardized reference for healthcare professionals. The emphasis on shared documents and platforms for policy dissemination ensures uniformity and consistency in the application of guidelines. Furthermore, creating an environment for active knowledge sharing among healthcare professionals is identified as a crucial facilitator. The reported debriefing sessions and collaborative approaches underscore the value of shared experiences and insights in promoting a cohesive approach to patient care. Regular check-ups play a crucial role in monitoring and managing the health status of individuals with comorbid conditions. The emphasis on proactive measures, such as calling patients for check-ups and encouraging medication adherence during travel, reflects a patient-centric approach. Lastly, motivational packages are identified as a pivotal facilitator in encouraging patient adherence to prescribed treatments and healthcare regimens. The reported initiatives, such as providing refreshments, organizing parties, and offering awards, highlight the importance of incentivizing positive health behaviors [54].

### Limitation of the study

The study included a relatively small sample size of 11 participants from three regions in Ghana, therefore the findings may lack generalizability to a larger population.

## Conclusion

The experiences of individuals with HIV and comorbidities profoundly impact their HRQoL, extending beyond medical complexities to emotional and financial dimensions. While the study highlights critical policies such as support groups, home visits, provision of free drugs, and counseling, it also underscores pervasive barriers, including non-adherence, stigma, financial constraints, and accessibility issues.

Managing multiple chronic illnesses alongside HIV induces fear, anxiety, and considerable burden. The facilitators identified, such as in-service training, common guidelines, knowledge sharing, external resources, regular check-ups, and motivational packages, provide actionable insights for improving policy implementation. The findings emphasize the need for tailored, patient-centered approaches and targeted interventions to ensure effective implementation and improved health outcomes for individuals with comorbid conditions.

## Recommendations

The study recommends the implementation and reinforcement of an integrated care approach for individuals living with HIV and comorbid conditions within the same healthcare framework. Also, the implementation of tailored adherence support programs that address the unique challenges faced by PLWHA.
